# Prerequisites for pore formation in foods

**DOI:** 10.1016/j.crfs.2026.101419

**Published:** 2026-04-28

**Authors:** R.G.M. van der Sman

**Affiliations:** Wageningen-Food & Biobased Research, Netherlands; Food Process Engineering, Wageningen University & Research, Netherlands

**Keywords:** Porous snacks, Multiphysics model, Design rules, State diagram

## Abstract

Puffing of starchy food materials during intensive heating processes is governed by a complex interplay between heat and mass transfer, phase transitions, and viscoelastic deformation of the biopolymer matrix. In this work, this pore formation is investigated using a cell model consisting of an initially small gas cavity surrounded by a deformable starch matrix. The model accounts for heat transfer, moisture transport, vapour generation, and viscoelastic relaxation, and is used to systematically explore pore growth and stabilisation under a wide range of processing conditions. Via the simulations we aim to derive the prerequisite for stable pore formation, i.e. crust formation, that can be used for later evaluation of oil-less frying or intensive drying processes. These prerequisites are expressed in terms of dimensionless numbers, related to process conditions and material properties.

The simulations demonstrate for pore formation requires superheating of the matrix, which leads to gas overpressure inside the pore, as expressed by the condition the external temperature exceeds the boiling point. However, stable pore structures are obtained only if a glassy crust forms sufficiently rapidly, meaning that the final state of the matrix should fall in the glassy region. The dynamics of pore growth are further controlled by the Deborah number, relating the viscoelastic relaxation time to the characteristic time scale for moisture removal. Optimal and stable puffing occurs for Deborah numbers of order unity, whereas excessively small or large values lead to unbounded pore growth or premature arrest of expansion, respectively.

By analysing the trajectories of the matrix state in a starch state diagram containing the boiling and glass transition lines, the mechanisms underlying pore growth, stabilisation, and collapse are elucidated. The identified criteria provide physically based guidelines for assessing and optimising oil-free frying and drying technologies such as air frying, microwave-assisted vacuum drying, and superheated steam drying.

## Highlights

• Multiphysics model predicts pore formation.
• Large deformation mechanics is required for the description.
• Three design rules for pore formation are presented.
• Simulation results can be understood via the state diagram.

## Graphical abstract

**Figure:**
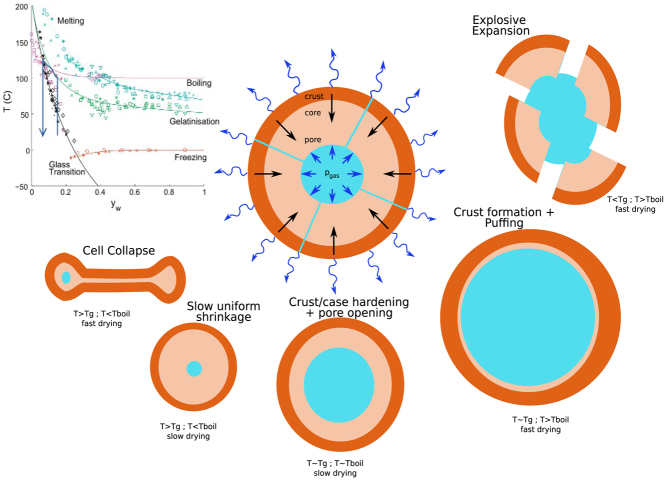

## 1 Introduction

In several food processing operations, gas-filled pores are deliberately induced to create a porous structure that is appealing to consumers (Scanlon and Koksel, 2024, Aghajanzadeh et al., 2024). Typical examples include intensive heating processes such as baking, frying, and puffing. Bubble formation generally relies on the generation of gas, most commonly steam produced from the moisture present in the food (Van der Sman and Broeze, 2014b, Van der Sman and Broeze, 2014a). In some cases, however, bubble formation depends on the release or dissolution of inert gases such as CO_2_ or N_2_, as observed in yeasted bread, leavened cakes and biscuits (Van Der Sman, 2021), or extruded snacks (Luo and Koksel, 2023).

To obtain a shelf-stable porous structure, the bubbles must be stabilised (Scanlon and Koksel, 2024). This stabilisation can occur through: (a) gelation of the biopolymeric matrix, for example via thermosetting of gluten or egg white proteins, or (b) transition of the biopolymeric matrix to a glassy state (Van der Sman and Broeze, 2014b, Van der Sman and Broeze, 2014a). Foods in the glassy state typically provide a crispy texture, whereas baked products such as cakes or bread, stabilised by a gelled protein network, exhibit a soft and fluffy texture.

Until recently, pore formation in foods was primarily described using empirical rules of thumb, with limited mechanistic understanding (Joardder et al., 2018, Purlis et al., 2021, Aghajanzadeh et al., 2024). In this paper, we present a mechanistic model that identifies the prerequisites for gas-filled pore formation in foods processed by intensive heating methods such as baking or frying. As such, this model builds upon our earlier work (Van der Sman and Broeze, 2014b, Van der Sman and Broeze, 2014a, Van der Sman et al., 2024).

We restrict our analysis to processes and products where bubbles are stabilised through the glass transition and where steam acts as the blowing agent, generated by moisture evaporation from the dough. Another constraint is that heat and mass transfer during the intensive heating process occurs via a gas phase, which may consist of air or steam, with heat transfer via conduction or convection, and do not consider volumetric heating as in radio-frequency of microwave. Consequently, our focus is on pore formation in processes such as baking, air-frying (impingement drying) (Moreira, 2001), explosion puffing (Swarnakar et al., 2022, Kaur et al., 2023), vacuum drying (Zhang et al., 2024), and superheated steam drying (Pimpaporn et al., 2007).

The key process control variables for these intensive heating operations are the external temperature ($T_{\text{ext}}$), external relative humidity ($RH_{\text{ext}}$), and ambient pressure ($p_{0}$). Heating increases the gas pressure inside the pores through internal evaporation of moisture. The extent of pore growth is governed by the balance between this internal gas pressure and the stress exerted by the expanding biopolymeric matrix. By altering the ambient pressure — as in vacuum drying — the boiling point of water within the food is effectively changed. Consequently, pore formation can occur at lower temperatures compared to atmospheric pressure.

An often-cited prerequisite for pore formation during intensive heating is crust formation at the product surface, also referred to as case-hardening in drying applications (Rahman, 2001, Van der Sman and Broeze, 2014b). Crust formation occurs when the food material approaches or enters the glassy state. This glass transition takes place at a characteristic temperature, $T_{g}$, which depends on the moisture content of the biopolymeric matrix. Consequently, the rheological properties of many food materials are strongly influenced by the ratio $T_{g}/T$ (Van der Sman and Mauer, 2019, Van der Sman et al., 2022, Van Der Sman et al., 2023), a parameter that will also be used in this study. Another prerequisite for significant pore growth, as observed in puffing, is that the moisture within the matrix must reach its boiling point (Van der Sman and Broeze, 2014b, Van der Sman and Broeze, 2014a).

Hence, in this study we are primarily interested in how pore formation is governed by the ratios $T_{g}/T_{\mathrm{ext}}$ and $T_{\mathrm{ext}}/T_{\mathrm{boil}}$, where $T_{\mathrm{ext}}$ is the external temperature imposed by the intensive heating process, $T_{g}$ is the glass transition temperature, which is a function of the moisture content $y_{w}$, and $T_{\mathrm{boil}}$ is the boiling temperature as determined by the external gas pressure $p_{0}$.

Ultimately, we aim to use these prerequisites for pore formation to evaluate intensive heating processes as alternatives to oil frying of French fries or expanded snacks, in which oil uptake is minimal while a crispy product can still be obtained. Often, oil frying process is regarded as the invasion of a boiling front (Farkas et al., 1996, Farid, 2001, Gouyo et al., 2021, Dehghannya et al., 2025). However, this picture is not consistent with viewpoints in poromechanics, when considering the solid matrix to be deformable. During drying air entry in the porous medium only occurs if a gas phase can be nucleated, or a pre-existing small gas pockets expands due to tensile (negative) pressures (Hu et al., 2013, Scherer and Smith, 1995). Cavitation preceeding meniscus invasion has been verified experimentally for aerogels via X-ray tomography (Gonthier et al., 2024). Hence, we assume this viewpoint to hold also for oil-frying of plant (potato) tissue, where pre-existing air pockets are present in the extracellular junctions between cells. These will expand at sufficiently large tensile stresses, generated by the stiffening of the crust, similar to our previous study (Van der Sman et al., 2024).

Processes alternative to oil-frying include vacuum frying, hot-air frying, and superheated steam frying (van Loon et al., 2005, Pimpaporn et al., 2007), as well as vacuum drying (Duarte-Correa et al., 2020), possibly assisted by infrared radiation, microwave heating, or ultrasound (Zhang et al., 2020, Dehghannya and Ngadi, 2021, Wang et al., 2022). Essentially, all these alternative processes can be regarded as drying processes, which becomes immediately apparent when comparing the boundary conditions used in simulation models for these processes (Farid, 2001, Halder et al., 2007, Warning et al., 2012, Van Koerten et al., 2017, Van der Sman et al., 2024). In that regard oil-frying is not much different than air-frying (Farid, 2001), only that the heat transfer coefficient is very high, and it evolves strongly with time, due to the variation of the vigorous motion of steam bubbles in the frying oil (Costa et al., 1999, Van Koerten et al., 2017).

Finally, we expect that the rates of heat and mass transfer are critical, as slow air frying or superheated steam drying may lead to structural collapse or case hardening of the tissue, thereby preventing further development of a crispy, porous crust (van Loon et al., 2005, Gazmuri and Bouchon, 2009, Gulati and Datta, 2015, Van der Sman and Schenk, 2024). We therefore follow the time-scale analysis proposed by Farid (2001).

We will investigate the governing rules for pore formation using a cell model. The first such cell model was developed by Amon and Denson (1984). In this model, a gas bubble grows in a viscous medium as a result of the balance between viscous stresses and gas pressure. Often, the cell model is supplemented with energy and mass balances for water. The Amon–Denson model has been used repeatedly to model pore formation in food applications, including starchy snack frying (Van der Sman and Broeze, 2014b, Van der Sman and Broeze, 2014a), supercritical CO${}_{2}$ snack extrusion (Alavi et al., 2003), directly extruded snacks (Fan et al., 1994, Wang et al., 2005, Manepalli et al., 2017), cheese (Laridon et al., 2020), and bread proofing (Hailemariam et al., 2007). In these models, the food matrix is regarded as a highly viscous fluid.

However, most food materials should be regarded as viscoelastic materials (Van der Sman et al., 2022, Sofou et al., 2008, Tariq et al., 1998, Ozturk and Takhar, 2020, Pinto and Takhar, 2023). Recently, we developed a model for pore formation that explicitly accounts for viscoelasticity (Van der Sman et al., 2024). In this paper we use this model as a cell model, and derive governing rules for pore formation. We assume the food to be spherical, with the material initially in the rubbery state. At the centre, a pre-existing minute pore is present, which is in equilibrium with the biopolymeric matrix. This pore will only expand to significant proportions under favourable conditions. We further assume that expansion occurs in closed pores.

In reality, at a later stage, pores may open and allow water vapour to escape; otherwise, the expanded structure would collapse upon cooling. This additional complexity, however, is excluded from the present simulations, which have the primary focus of establishing the pre-conditions for pore-formation. In the field of chemical engineering several models are presented, that deal with more complex that describe phenomena like coalescence and disproportionation (Everitt et al., 2006, Mitrias et al., 2019, Ataei et al., 2021, Tammaro et al., 2022), but they were often limited to either 2D simulations, or more simplified rheological models.

In this paper, we first provide a brief description of the model and subsequently present exemplary simulation results, each having distinct outcomes regarding pore formation. These results are depicted in a state diagram that includes both the boiling line and the glass transition line. This diagram illustrates how the ratios $T_{g}/T$ and $T/T_{\mathrm{boil}}$ evolve in the crust and pore regions, and how these ratios control pore formation. We conclude by formulating the governing rules for pore formation.

## 2 Model description

The governing equations are given for the reference frame, co-moving with the deforming polymer network. Hence, the time derivative is in terms of the material derivative: $D_{t}=\partial_{t}+\nabla \cdot v_{s}$, with $v_{s}$ the velocity of the solid phase.

The mass balance for the water in the core and shell is:

(1) $$D_{t}\phi_{w}=-\nabla \cdot j_{w}=\nabla \cdot \frac{D_{s}\nu_{w}}{R_{gas}T}\nabla \mu_{w}$$

$\phi_{w}$ is the volume fraction of water, $j_{w}$ is the diffusive water flux given by generalised Fick’s law, $D_{s}$ is the self-diffusivity of water, $\nu_{w}$ is the molar volume of water, $R_{gas}$ is the universal gas constant, and $\mu_{w}$ is the chemical potential.

The chemical potential will have two contributions, one due to the mixing energy of water and biopolymers, and another due to the elastic energy due to the deformation (Hong et al., 2009):

(2) $$\mu_{w}=\mu_{w,mix}+p_{liq}=-\Pi_{mix}+p_{liq}$$

The osmotic pressure $\Pi_{mix}$ is the mixing contribution, and $p_{liq}$ is the hydrostatic pressure in the solvent, resulting from the deformation. The mixing contribution follows the Flory–Huggins theory, with a composition dependent interaction parameter (Jin and van der Sman, 2022):

(3) $$\mu_{w,mix}\nu_{w}/RT=\log\left(\phi_{w}\right)+\left(1-\phi_{w}\right)+\chi_{eff}{\left(1-\phi_{w}\right)}^{2}$$

Recall, the polymer volume fraction is $\phi_{s}=1-\phi_{w}$. The interaction parameter is:

(4) $$\chi_{eff}=\chi_{0}+\left(\chi_{1}-\chi_{0}\right){\left(1-\phi_{w}\right)}^{2}$$

$\chi_{0}=0.5$ is the universal value for hydrated biopolymers. $\chi_{1}$ depends on the particular biopolymers, and it is expected to be in the range ($0.8\leq \chi_{1}\leq 1.4$) (Van der Sman and Meinders, 2011, Van der Sman, 2012).

During dehydration/swelling of soft matter, one commonly assumes that the momentum balance is in a steady state (Hong et al., 2009):

(5) ∇⋅σ=0

σ is the stress tensor, which follows from the thermodynamic theory of Suo (Hong et al., 2009). For spherical geometries, the stress has only non-zero diagonal components, $\sigma_{rr}$, and $\sigma_{\theta \theta }=\sigma_{\phi \phi }$, which are formulated in terms of the stretches $\lambda_{i}$ of the polymer network in the principal directions. The stretch parameters are related via the incompressibility condition:

(6) $$\overset{\sim }{\phi }\lambda_{r}\lambda_{\theta }^{2}=1$$

It is convenient to define the swelling factor $J=\lambda_{r}\lambda_{\theta }^{2}$.

We follow the Neo-Hookean model for the stress components (Van der Sman, 2015). For pure elastic materials, the stress follows:

(7) $$\sigma_{ii}=\overset{\sim }{\phi }G_{0}\lambda_{i}^{2}-p_{liq}$$

Below, we will give the stress relations for viscoelastic materials.

The energy balance reads (Li, 2009):

(8) $$D_{t}C_{eff}T=-\nabla \cdot Q$$

$C_{eff}$ is the volumetric heat capacity, which changes due to moisture transfer (Li, 2009). Hence,

(9) $$C_{eff}=\phi_{w}C_{w}+\phi_{s}C_{s}$$

with $C_{i}$ the volumetric heat capacity of each phase.

The heat flux Q follows (Moya et al., 2021):

(10) $$Q=-k\nabla T+C_{w}j_{w}T$$

The first contribution accounts for heat conduction, and the second contribution accounts for the convection of heat by the water flow, which has the volumetric heat capacity $C_{w}$. We assume that the thermal conductivity k is a simple volume average (Van der Sman, 2008):

(11) $$k=\phi_{w}k_{w}+\phi_{s}k_{s}$$

$k_{i}$ is the thermal conductivity of each phase.

For the viscoelastic relaxation, we follow Bosnjak et al. (2020), where the deformation gradient is decomposed in an elastic and viscous contribution:

(12) $$\lambda_{i}=\lambda_{i,e}\lambda_{i,v}$$

It is assumed that $J_{v}=\lambda_{\theta ,v}^{2}\lambda_{r,v}=1$. Hence, we can define the internal variable:

(13) $$A=1/\lambda_{\theta ,v}^{2}=\lambda_{r,v}$$

from which follows:

(14) $$\lambda_{\theta ,e}^{2}=\lambda_{\theta }^{2}/A$$

The internal variable evolves following a single relaxation time scheme:

(15) $$\frac{dA}{dt}=\frac{1}{\tau_{visco}}\left(\frac{1}{\lambda_{\theta }^{2}}-A\right)$$

with $\tau_{visco}$ the viscoelastic relaxation time.

The principal components of the stress can be rewritten as:

$$\sigma_{\theta \theta }=\frac{G_{0}}{J}\lambda_{\theta }^{2}A-p$$

(16) $$\sigma_{rr}=\frac{G_{0}}{J}\frac{1}{\lambda_{\theta }^{4}A^{2}}-p$$

### 2.1 Boundary conditions

For the mass balance, we apply Robin-type boundary conditions. At the inner boundary, at $r=r_{in}$, the following relation holds:

(17) $$j_{w}\cdot \overset{ˆ}{n}\left(r_{in}\right)=-j_{cav}=-\frac{\beta_{cav}}{M_{w}}\left[a_{w}\left(r_{in}\right)-RH_{cav}\right]c_{sat}\left(T_{gas}\right).$$

Here, $a_{w}\left(r_{in}\right)$ is the water activity, which is related to the chemical potential of the hydrogel at the inner boundary via $\mu_{w}\left(r_{in}\right)=R_{gas}T_{p}\log\left(a_{w}\right)$. $RH_{cav}$ is the relative humidity of the gas inside the pore, related to the vapour pressure through $RH_{cav}=p_{vap}/p_{sat}\left(T_{gas}\right)$, where $p_{sat}\left(T\right)$ is the saturated vapour pressure at temperature T. The gas temperature is assumed to be equal to the local product temperature, i.e. $T_{gas}=T_{p}\left(r_{in}\right)$. The saturated vapour concentration is given by $c_{sat}\left(T\right)=M_{w}p_{sat}\left(T\right)/\left(R_{gas}T\right)$ (in kg/m${}^{3}$). $M_{w}$ denotes the molar mass of water. The mass transfer coefficient for water transport inside the pore is taken as $\beta_{cav}=D_{air}/\left(r_{in}/5\right)$ (Van der Sman et al., 2024).

At the outer boundary, at $r=r_{out}$, the mass flux is given by

(18) $$j_{w}\cdot \overset{ˆ}{n}\left(r_{out}\right)=+j_{evap}=\frac{\beta_{air}}{M_{w}}\left[a_{w,ext}\;c_{sat}\left(T_{p}\right)-RH_{ext}\;c_{sat}\left(T_{ext}\right)\right].$$

Here, $\beta_{air}=h_{air}/\left(\rho_{air}c_{p,air}\right)$ is the mass transfer coefficient of the external boundary layer. Following the Lewis relation, it is linearly related to the heat transfer coefficient of the boundary layer, $h_{air}$. The quantity $\rho_{air}c_{p,air}$ denotes the volumetric heat capacity of air. The water activity at the outer surface is defined by $\mu_{w}\left(r_{out}\right)=R_{gas}T_{p}\log\left(a_{w,ext}\right)$. The external relative humidity, $RH_{ext}$, and the drying-air temperature, $T_{ext}$, are process parameters of the drying operation.

For the momentum balance, we impose pressures at the outer and inner boundary:

(19) $$\sigma_{rr}\left(r_{out}\right)=p_{0}\text{;}\sigma_{rr}\left(r_{in}\right)=p_{gas}$$

The gas pressure $p_{gas}$ is the sum of the partial air and water pressures:

(20) $$p_{gas}=p_{air}+p_{vap}$$

As the number of moles of air $N_{air}$ remains constant we have:

(21) $$N_{air}=\frac{p_{air,0}V_{gas,0}}{R_{gas}T_{init}}=\frac{p_{air}V_{gas}}{R_{gas}T_{gas}}$$

with $p_{air,0}=p_{0}-a_{w}p_{sat}\left(T_{init}\right)$ the initial air pressure, $V_{gas,0}=4/3\pi R_{in}^{3}$ is the initial pore volume, $T_{init}$ is the initial product temperature, $V_{gas}=4/3\pi r_{in}^{3}$ is the actual pore volume. $R_{in}$ is the initial pore radius, and $p_{0}$ is the ambient pressure.

The partial water vapour pressure follows:

(22) $$p_{vap}V_{gas}=N_{vap}R_{gas}T_{gas}$$

with

(23) $$\frac{dN_{vap}}{dt}=j_{cav}4\pi r_{in}^{2}$$

which will be integrated during simulations to obtain $N_{vap}$.

The boundary condition for the heat flux at the outer boundary is:

(24) $$Q\cdot \overset{ˆ}{n}\left(r_{out}\right)=+Q_{evap}=h_{air}\left(T_{p}-T_{ext}\right)+j_{evap}\Delta H_{evap}$$

$\Delta H_{evap}$ is the heat of evaporation, expressed in J/mol.

At the inner boundary we assume the gas temperature equal to the product temperature, and hence:

(25) $$\partial_{r}T_{p}\left(r_{in}\right)=0$$

### 2.2 Implementation

The model is implemented using the finite element method in COMSOL, employing the weak formulation. The food material is assumed to be spherical, and the problem is solved in one spatial dimension along the radial coordinate. For the interested reader, the weak forms of the governing equations can be found in our previous paper (Van der Sman et al., 2024). The initial state is stress-free (fully relaxed), implying that $\lambda_{i}\left(t=0\right)=1$.

The diffusion coefficient follows free-volume theory, as explained in our earlier work (Van der Sman and Meinders, 2013). It should be noted that the diffusion coefficient changes over several orders of magnitude during drying.

The viscoelastic relaxation time, $\tau_{\mathrm{visco}}=\eta /G_{\infty }$, is assumed to depend on the ratio of the viscosity to the elastic modulus in the glassy state, with $G_{\infty }=1\;\mathrm{GPa}$. The viscosity is a function of $T_{g}/T$ (Van der Sman et al., 2022), where $T_{g}$ denotes the glass transition temperature. We assume that the glass transition of the food material follows the Couchman–Karasz relation (Van der Sman et al., 2024). The viscosity follows the relation reported for maltodextrins (Van der Sman et al., 2022). The elastic modulus in the rubbery regime is taken as $G_{0}=1\;\mathrm{MPa}$.

To represent a cell model, the radius of the spherical food object is chosen as $R_{out}=1\;\mathrm{mm}$, which also resembles the size of micropellets used for starchy snack expansion (Van der Sman, 2016). The initial gas bubble radius is set to $R_{in}=0.05\;R_{out}$. The initial polymer volume fraction, $\phi_{s,0}>0$, is varied in the parameter studies, implying that $a_{w}<1$ in the initial state. In addition, we vary the ambient pressure $p_{0}$, the external temperature $T_{ext}$, the relative humidity of the hot air $RH_{ext}$, and the heat transfer coefficient $h_{air}$. Through appropriate choices of $T_{ext}$ and $RH_{ext}$, we can control whether the food material enters the glassy state. As the Flory–Huggins interaction parameter is temperature independent (Van der Sman and Meinders, 2011), $RH_{ext}$ directly determines the final water activity at the end of the drying process.

### 2.3 State diagram generation

We analyse the simulation results using a state diagram in which both the glass transition line and the boiling line are depicted. In addition, the evolution of the product state is plotted in this diagram, characterised by the temperature T and moisture content $y_{w}$ at the inner and outer surfaces of the biopolymer matrix surrounding the expanding pore. Of particular interest is the path followed by the product state relative to the boiling and glass transition lines, as expressed by the ratios $T_{boil}/T$ and $T_{g}/T$. In this section, we describe the construction of these lines. We follow the construction of the starch state diagram presented in our earlier work (Van der Sman and Meinders, 2011).

The glass transition line is described by the Couchman–Karasz relation:

(26) $$T_{g}=\frac{y_{w}\Delta c_{p,w}T_{g,w}+\left(1-y_{w}\right)\Delta c_{p,s}T_{g,s}}{y_{w}\Delta c_{p,w}+\left(1-y_{w}\right)\Delta c_{p,s}}$$

Here, $T_{g,i}$ denotes the glass transition temperature of component i (with i=s for starch and i=w for water), and $\Delta c_{p,i}$ is the change in specific heat capacity at the glass transition of the pure component. The variable $y_{w}$ represents the mass fraction of water, and $T_{g}$ is the overall glass transition temperature as a function of $y_{w}$. The parameter values used in this relation are reported in Van der Sman and Meinders (2011).

Starch/water mixtures are assumed to follow the Flory–Huggins theory for moisture sorption, rendering the water activity $a_{w}$ (Van der Sman and Meinders, 2011). Using Clausius–Clapeyron, we compute the elevation of the boiling temperature $T_{boil}$ at lower water activities $a_{w}$:

(27) $$\frac{1}{T_{boil}}=\frac{1}{T_{boil,0}\left(p_{0}\right)}+\frac{R_{gas}}{\Delta H_{evap}}\log\left(a_{w}\right)$$

$T_{boil,0}\left(p_{0}\right)$ is the boiling point of pure water at standard pressure $p_{0}$=100 kPa. The boiling point is also controlled via the ambient pressure $p_{ext}$, which also follows from the Clausius–Clapeyron relation:

(28) $$\frac{p_{ext}}{p_{0}}=\exp\left[-\frac{\Delta H_{evap}}{R_{gas}}\left(\frac{1}{T_{boil}\left(p_{ext}\right)}-\frac{1}{T_{boil}\left(p_{0}\right)}\right)\right]$$

The water activity follows Flory–Huggins:

(29) $$\log\left(a_{w}\right)=\log\left(\phi_{w}\right)+\left(1-\phi_{w}\right)+\chi_{eff}{\left(1-\phi_{w}\right)}^{2}$$

with the composition dependent Flory–Huggins interaction parameter:

(30) $$\chi_{eff}=\chi_{0}+\left(\chi_{1}-\chi_{0}\right){\left(1-\phi_{w}\right)}^{2}$$

$\chi_{0}=0.5$ holds universally for biopolymers, and $\chi_{1}$ depends on the particular biopolymer. For starch it holds $\chi_{1}=0.8$ (Van der Sman and Meinders, 2011).

### 2.4 Time scale analysis

We determine the time scale for the formation of a crust of a given thickness during frying and compare it with the time scale for heat transfer, following the approach of Farid (2001). The total mass flux at the surface, $J_{evap}$, is linear in the difference in vapour pressure, $p_{vap,p}-p_{vap,ext}$, while the heat flux, $Q_{evap}$, is linear in the temperature difference between the product and the frying medium $\left(T_{p}-T_{ext}\right)$, minus the heat required for evaporation:

$$J_{evap}∼\beta A\left(p_{vap,p}-p_{vap,ext}\right)/\left(RT_{p}\right),$$

(31) $$Q_{evap}∼hA\left(T_{p}-T_{ext}\right)-\Delta H_{evap}J_{evap}.$$

Here, β is the mass transfer coefficient, h the heat transfer coefficient, A the surface area, R the gas constant, and $\Delta H_{evap}$ the latent heat of evaporation. For air drying or air frying, β and h are related through the Lewis relation. In our simulations we take heat transfer relevant to air-frying, h=100 W/m${}^{2}$ K.

During oil frying, the product temperature rapidly reaches the boiling temperature, $T_{p}=T_{boil}$, which depends on the ambient pressure. Moreover, during oil frying $p_{vap,ext}$ is taken equal to the ambient pressure $p_{0}$, since the saturated vapour pressure at the boiling point equals $p_{0}$. Similar relations apply to vacuum frying (Sobukola et al., 2013), where the boiling point is reduced due to the lower pressure. Frying assisted by microwave heating can also be interpreted within this framework, as volumetric heating effectively increases the vapour pressure $p_{vap,p}$ (Teleken et al., 2021).

The majority of the moisture is removed from the food product during the first stage of drying or frying, in which the product temperature attains a constant value $T_{wb}$ (the wet-bulb temperature or boiling temperature) and the water activity remains unity. In this quasi-steady state, the supplied heat is fully used for moisture evaporation:

(32) $$hA\left(T_{wb}-T_{0}\right)=\Delta H_{evap}\;\beta A\;\frac{p_{v}ap\left(T_{wb}\right)-p_{vap,ext}}{RT_{p}}.$$

The frying process should create a crust of a certain thickness, implying that a fraction ϕ of the total moisture is removed, i.e. $\Delta m_{w}=\phi \rho_{w}V$. The total required frying time then follows as

(33) $$t_{e}=\frac{\Delta m_{w}}{J_{evap}}=\frac{\phi \rho_{w}V\Delta H_{evap}}{hA\left(T_{wb}-T_{ext}\right)}.$$

Using the time scale for heat transfer:

(34) $$\tau_{RC}=\left(\rho_{w}cp_{w}V\right)/\left(hA\right)$$

the expression In dimensionless form becomes:

(35) $$\frac{t_{e}}{\tau_{RC}}=\frac{\phi \Delta H_{evap}}{c_{p,w}\left(T_{wb}-T_{ext}\right)}.$$

The term $\phi \Delta H_{evap}/\left[c_{p,w}\left(T_{wb}-T_{0}\right)\right]$ is equal to 1/Ste, where Ste denotes the Stefan number (Farid, 2001). Other relevant dimensionless numbers are the Fourier and Biot numbers:

$$Fo=\frac{k}{\rho_{w}c_{p,w}}\frac{t_{e}}{L^{2}},$$

(36) $$Bi=\frac{hL}{k}.$$

The Fourier number represents the ratio of the process time to the characteristic time scale for heating the product by conduction, with $k/\left(\rho_{w}c_{p,w}\right)$ being the thermal diffusivity. The Biot number expresses the ratio of internal to external thermal resistance. The characteristic length scale is defined as L=V/A, where V is the volume and A the surface area. Thus, we can also rewrite:

(37) $$\frac{\tau_{RC}}{t_{e}}=\frac{\rho_{w}c_{p,w}L^{2}}{kt_{e}}\frac{k}{hL}=\frac{1}{FoBi}$$

Achanta et al. (1997) further state that the Deborah number plays an important role in pore formation, as it expresses the ratio between the viscoelastic relaxation time and the characteristic time scale for moisture removal. Accordingly, we define

(38) $$De=\frac{\tau_{\text{visco}}}{t_{e}}=\tau_{\text{visco}}\left(T_{g}/T\right)\;\frac{hA\;\left(T_{p}-T_{ext}\right)}{\phi \;\rho_{w}V\;\Delta H_{\text{evap}}}.$$

Here, $\tau_{\text{visco}}\left(T_{g}/T\right)$ is the viscoelastic relaxation time, which depends on the ratio $T_{g}/T$, and is given by (Van der Sman et al., 2024):

(39) $$\tau_{\text{visco}}\left(T_{g}/T\right)=\frac{\eta \left(T_{g}/T\right)}{G_{\infty }},$$

where η is the viscosity of the biopolymeric matrix and $G_{\infty }$ is the elastic modulus of the matrix in the glassy state. The viscosity follows the relation determined for maltodextrins, as described in Van der Sman et al. (2022).

Due to the continuous loss of moisture and the concurrent change in product temperature $T_{p}$, the Deborah number varies over a wide range during intensive heating. In the following, we therefore investigate the temporal evolution of De and its relation to pore development.

We note that in the simulations we often used a high initial moisture content (70%) - comparable to French fries, which is considerably higher than the initial moisture content of expanded snacks (14%–20%). This will make the process time $t_{e}$ in the order of 200–300 s, as excess moisture needs to be removed. Often, simulations are performed with final times $t_{end}=1000$ s, in order to show whether true steady state is reached. Practical process times ($t_{e}$) will be shorter that (in order of 100 s). But alltogether, the relative scale of $t_{e}$ compared to the viscoelastic relaxation time (the Deborah number) is what matters.

## 3 Simulations

### 3.1 Exemplary pore formation scenarios

To gain insight into the pore formation process, we start with exemplary simulations illustrating several typical pore formation scenarios. We present the temporal evolution of the relevant state variables of the system and represent the product state in the $\left(y_{w},T\right)$ plane of the starch state diagram, including the glass transition and boiling lines.

**Fig. 1:**
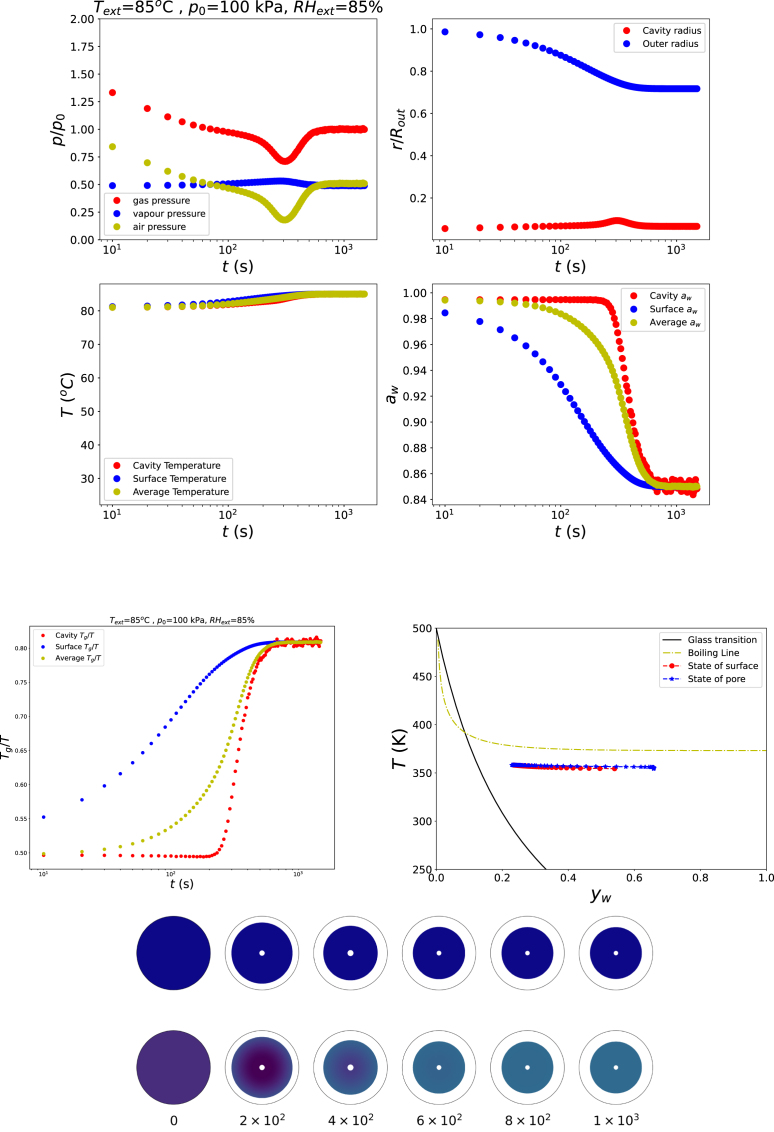
Changes in states of food product in the rubbery state, with $T_{ext}=85\;{}^{\circ}C$, $RH_{ext}=85\text{\%}$, and $p=p_{0}$.

We first consider a simulation in which the material remains entirely in the rubbery state. The external relative humidity is set to $RH_{\text{ext}}=0.85$, the ambient pressure to $p_{0}=100\;\text{kPa}$, and the air temperature is chosen below the boiling point, $T_{\text{ext}}=85\;{}^{\circ}\text{C}$. The initial polymer volume fraction is $\phi_{s,0}=0.25$. Results are prensented in Fig. 1.

The results of all following simulation runs will be presented in a similar manner, as will be explained below. The top panes of the figure indicates (a) the evolution of the gas pressure ($p/p_{0}\left(t\right)$), (b) the changes in cavity radius and outer product radius ($r_{in}/R_{out}\left(t\right)$ and $r_{out}/R_{out}\left(t\right)$, (c) the average, surface, and cavity temperatures as function of time ($\overset{¯}{T}\left(t\right),T_{out}\left(t\right),T_{in}\left(t\right)$), and (d) the average, surface, and cavity water activity as function of time (${\overset{¯}{a}}_{w}\left(t\right),a_{w,out}\left(t\right),a_{w,in}\left(t\right)$), (e) the evolution of the ratio $T_{g}/T$ (on average, surface and cavity), together with the water activity $a_{w}$. The states of the outer surface and pore wall (cavity wall), in terms of temperature (T) and moisture content ($y_{w}$), are depicted in the state diagram, also showing the boiling line, and glass transition line - to observe if conditions $T_{g}/T>1$ or $T/T_{boil}>1$ are occuring. Furthermore, the bottom panes are showing the radial profiles of the ratio $T_{g}/T$ and the radial stress $\sigma_{rr}$ depicted as contour plots at equidistant time intervals, in the co-moving coordinate frame, illustrating the spatio-temporal development of thermal, rheological, and mechanical gradients within the product.

Fig. 1 illustrates the behaviour, if the food remains in the rubbery state ($T_{g}/T<1$) and its temperature does not exceed the boiling point, namely little structural change occurs. These results are obtained from a simulation with $RH_{\text{ext}}=85\text{\%}$, $T_{\text{ext}}=85\;{}^{\circ}\text{C}$, and $p=p_{0}$. The simulation shows that the product predominantly shrinks. As a consequence of dehydration, stresses develop in the outer skin, which give rise to a slight and temporary increase in the cavity radius. However, these stresses relax rapidly, leading to a subsequent decrease of the cavity size.

**Fig. 2:**
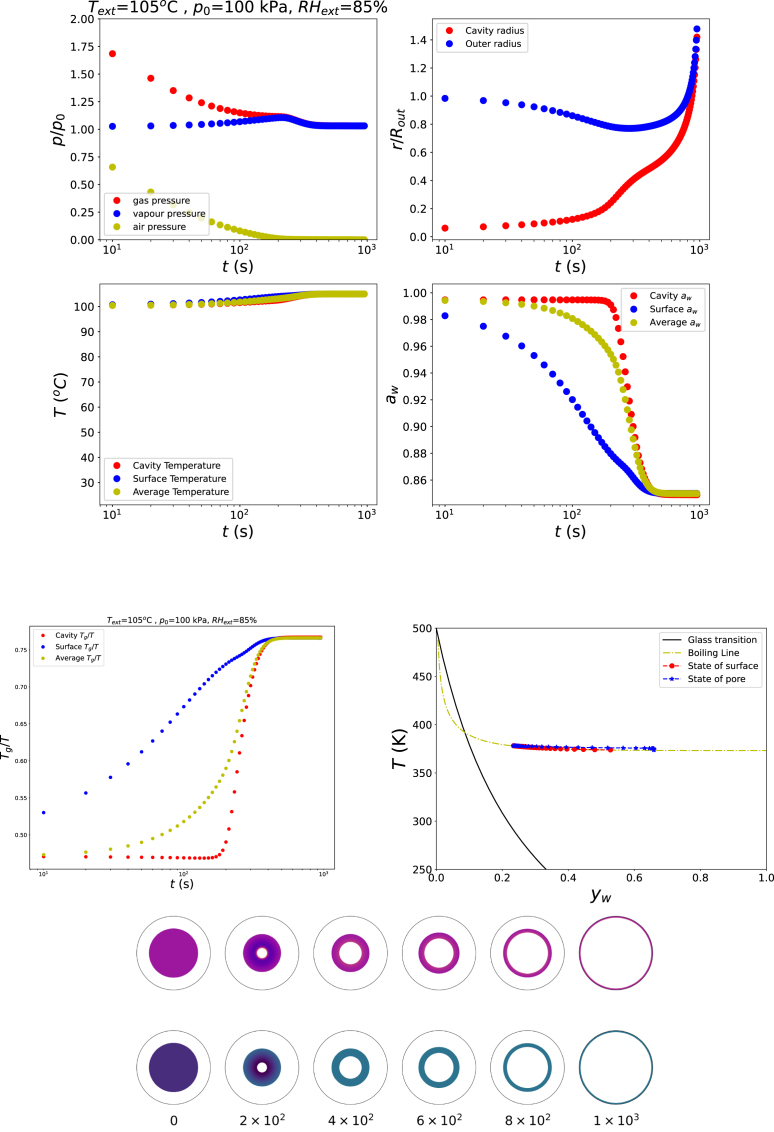
Changes in states of food product in the rubbery state, with temperature exceeding boiling point $T_{ext}=105\;{}^{\circ}C$, $RH_{ext}=85\text{\%}$, and $p=p_{0}$.

If the food remains in the rubbery state ($T_{g}/T<1$) while the product temperature exceeds the boiling point, an explosive pore growth is observed, as shown in Fig. 2. These simulations are performed under conditions $RH_{\text{ext}}=105\text{\%}$, $T_{\text{ext}}=85\;{}^{\circ}\text{C}$, and $p=p_{0}$. From the starch state diagram, it follows that the cavity state closely follows the boiling line during dehydration. The resulting vapour overpressure inside the cavity drives rapid pore expansion, while dehydration simultaneously causes shrinkage of the surrounding biopolymeric shell. Because the shell remains in the rubbery state, the viscoelastic relaxation times are short and no significant stresses develop to counteract the gas-driven expansion. In practice, the food matrix cannot sustain such large deformations and mechanical rupture of the shell is expected to occur.

**Fig. 3:**
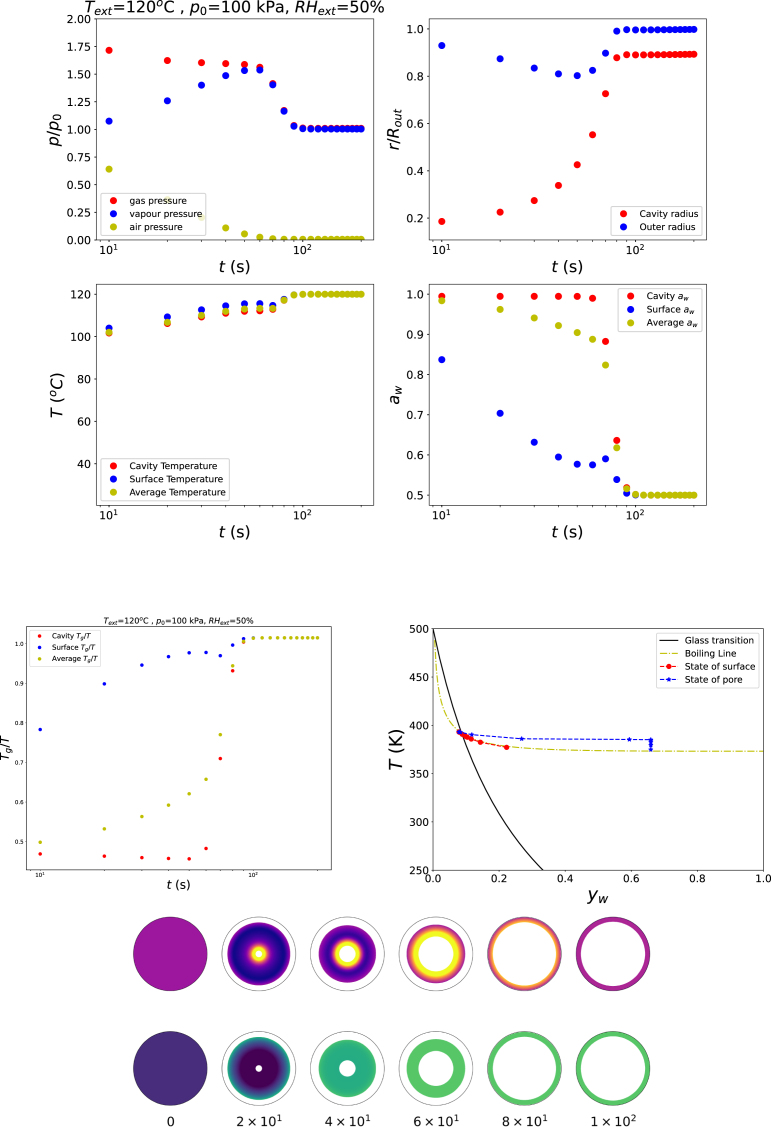
Changes in states of food product in the rubbery state, but ending in the glassy state with $T_{ext}=120\;{}^{\circ}C$, $RH_{ext}=50\text{\%}$, and $p=p_{0}$.

When the external relative humidity is reduced to $RH_{\text{ext}}=50\text{\%}$, while the temperature is raised above the boiling point ($T_{\text{ext}}=120\;{}^{\circ}\text{C}$ at $p=p_{0}$), the dehydrating shell rapidly transitions from the rubbery state to the glassy state. This transition allows the pore to grow initially, but subsequently stabilises at a finite size once the entire shell has become glassy. This behaviour is illustrated in Fig. 3. The corresponding path in the state diagram shows that the shell surface follows the boiling line, whereas the shell material surrounding the pore deviates from it due to the elevated gas pressure relative to the external pressure. Towards the end of the drying process, as the water activity at the inner surface deviates from unity, the gas pressure decreases and approaches $p_{\text{gas}}\approx p_{0}$.

**Fig. 4:**
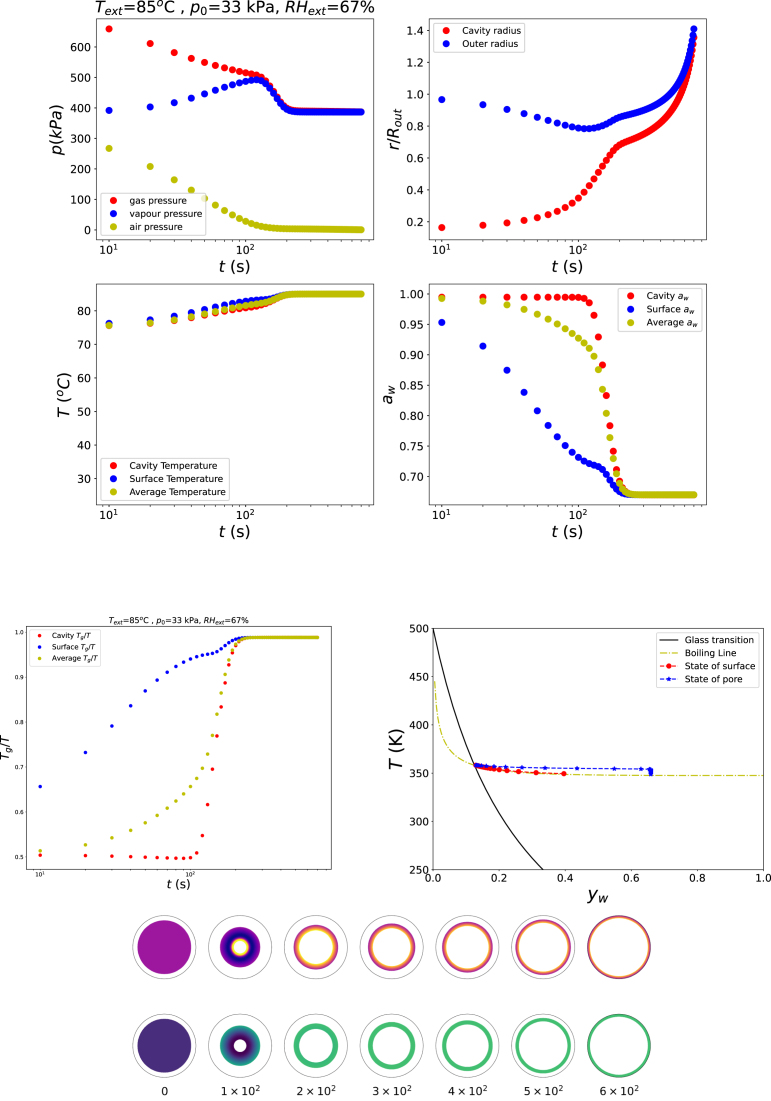
Changes in states of food product in the rubbery state, ending in the glassy state, boiling at lowered ambient pressure: $T_{ext}=85\;{}^{\circ}C$, $RH_{ext}=67\text{\%}$, and $p=\frac{1}{3}p_{0}$.

Pore growth can also occur at temperatures below 100 °C when the ambient pressure is reduced. This is illustrated in Fig. 4, which presents simulation results for $T_{\text{ext}}=85\;{}^{\circ}\text{C}$, $RH_{\text{ext}}=67\text{\%}$, and $p=\frac{1}{3}p_{0}$. The corresponding path in the starch state diagram shows that the outer surface of the shell follows the boiling line, whereas the inner surface deviates from it due to the elevated gas pressure inside the bubble, similar to the behaviour observed in the previous simulations (see Fig. 3). However, the final state of the shell remains just below the glass transition. As a result, the material cannot effectively resist the gas-driven expansion, and explosive growth of the bubble is not prevented. This observation highlights the importance of reaching a final drying state that is well within the glassy regime, with $T_{g}/T>1$, in order to stabilise the pore structure.

**Fig. 5:**
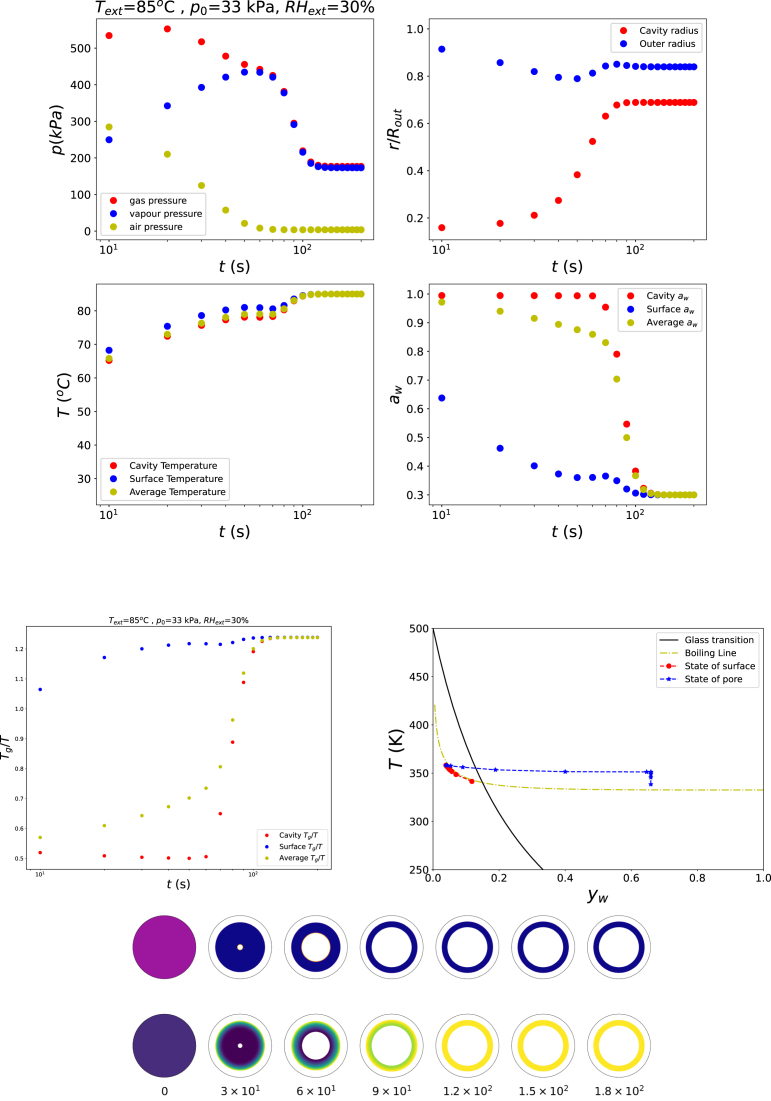
Changes in states of food product in the rubbery state, but quickly entering glassy state, boiling at low pressure: $T_{ext}=85\;{}^{\circ}C$, $RH_{ext}=30\text{\%}$, and $p=\frac{1}{3}p_{0}$.

For the next simulation, the final state is driven deep into the glassy regime by reducing the external relative humidity to $RH_{\text{ext}}=30\text{\%}$, while keeping $T_{\text{ext}}=85\;{}^{\circ}\text{C}$ and $p=\frac{1}{3}p_{0}$. The results shown in Fig. 5 confirm that pore growth occurs and subsequently stabilises at a finite size. The corresponding state-diagram trajectory indicates that the outer surface of the shell rapidly enters the glassy state. This glassy skin inhibits further shrinkage of the overall cell system. Nevertheless, the pore continues to grow for some time due to ongoing dehydration of the shell material, until the fully glassy structure arrests further deformation.

### 3.2 Parameter studies

**Fig. 6:**
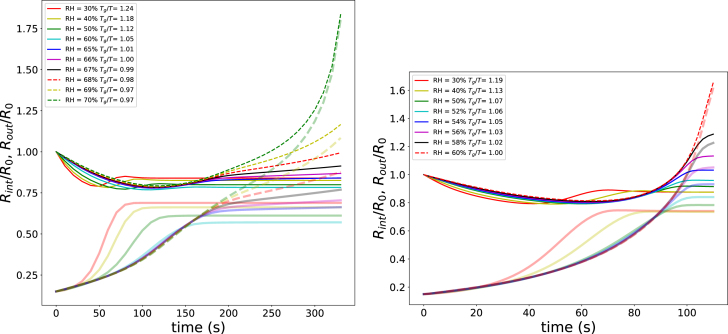
Growth of inner (translucent lines) and outer radius (solid lines) of the unit cell as function of $RH_{ext}$ at (a) $T_{ext}=85\;{}^{\circ}C$, and $p_{ext}=\frac{1}{3}p_{0}$ (left pane), and (b) $T_{ext}=100\;{}^{\circ}C$, and $p_{ext}=\frac{1}{3}p_{0}$ (right pane). In the legend values of $T_{g}/T$ of matrix corresponding with final state at $T=T_{ext}$ and $a_{w}=RH_{ext}$.

In this section, we vary several model parameters to verify that pore formation is governed by the ratios $T_{g}/T$, $T/T_{\text{boil}}$, and the Deborah number De. First, we investigate the conditions for optimal pore expansion at $T_{\text{ext}}=85$ and 100 °C and an external pressure of $p_{\text{ext}}=\frac{1}{3}p_{0}$, for a range of relative humidities $30\text{\%}<RH_{\text{ext}}<67\text{\%}$. The corresponding simulation results are shown in Fig. 6.

We observe that when $T_{g}/T<1$ — based on the final state of the product defined by $a_{w}=RH_{\text{ext}}$ and $T=T_{\text{ext}}$ — the pore radius increases without bound. In contrast, a stable porous structure is formed when $T_{g}/T>1$, as the pore radius grows to a finite, stable value. Maximum expansion is obtained in the transition regime, where $T_{g}/T\approx 1$.

The degree of expansion further depends on the ratio $T/T_{\text{boil}}$, which quantifies the level of superheating. For the initial water mass fraction $y_{w,0}=0.65$, the corresponding water activity and boiling temperature were computed at $p_{\text{ext}}=\frac{1}{3}p_{0}$. This yields $T/T_{\text{boil}}=1.08$ for $T_{\text{ext}}=85\;{}^{\circ}\text{C}$ and $T/T_{\text{boil}}=1.12$ for $T_{\text{ext}}=100\;{}^{\circ}\text{C}$. Under the latter conditions, the simulations predict puffed structures with dimensions exceeding those of the initial wet sphere.

**Fig. 7:**
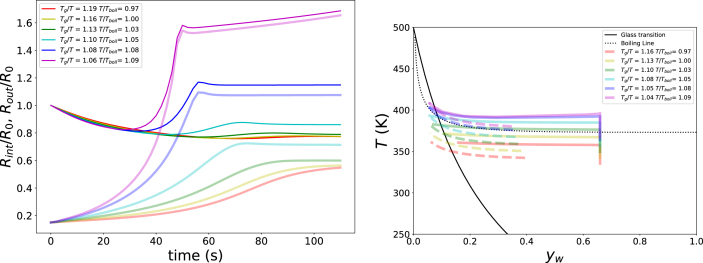
Left pane: Growth of inner (translucent lines) and outer radius (solid lines) of the unit cell as function of $T_{ext}$ at $RH_{ext}=40$%, $p_{ext}=p_{0}$, and $y_{w,0}=0.65$. The legend states values of $T_{g}/T$ and $T_{boil}/T$. Right pane: Corresponding paths of the outer surface (dashed lines) and pore wall (solid lines) in the state diagram.

Next, we explored the pore formation as function of $T_{ext}$, while keeping $RH_{ext}=40$%, $p_{ext}=p_{0}$, and $y_{w,0}=0.65$. Results are shown in Fig. 7. If $T_{ext}/T_{boil}\leq 1.0$ the outer radius only decreases in size, and quickly becomes stable due to the formation of a glassy crust. The inner pore only grows due to the loss of moisture. Only, if $T_{ext}/T_{boil}>1.03$ there is sufficient gas pressure build up for puffing: after initial decrease, the outer radius increases again. This is especially dramatic for $T_{ext}/T_{boil}=1.09$, where $T_{g}/T_{ext}=1.06$. Hence, only at a late stage the outer shell gets into the glassy state, and the system pufs beyond its original size. However, if we increase $T_{ext}$ further, the bubble grows unboundedly - due to $T_{g}/T_{ext}\approx 1.0$. Thus at high initial moisture content, there is only a small window of $T_{ext}/T_{boil}$ where stable (puffed) pore formation happens.

When plotting the simulated states in the state diagram of Fig. 7, one observes confirmation of the above conclusions. If $T_{ext}/T_{boil}>1$, the path of the inner surface is above the boiling line, indicating generation of excess gas pressures $p_{gas}>p_{0}$. Further increase of $T_{ext}$, further increases the path above the boiling line, however the outer surface gets into the glassy state much later - as indicated by the fact that only the last part of the path gets in the glassy regime, stabilising the pore. Eventually $T_{g}/T_{ext}<=1$, and there will be unbounded pore growth.

**Fig. 8:**
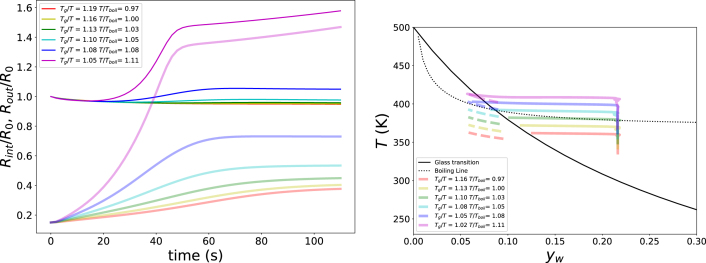
Left pane: Growth of inner (translucent lines) and outer radius (solid lines) of the unit cell as function of $T_{ext}$ at $RH_{ext}=40$%, $p_{ext}=p_{0}$, and $y_{w,0}=0.22$. The legend states values of $T_{g}/T$ and $T_{boil}/T$. Right pane: Corresponding paths of the outer surface (dashed lines) and pore wall (solid lines) in the state diagram.

In the next series of simulations, we examine whether the puffing window is broadened at lower initial moisture content. The simulations are performed for an initial water mass fraction of $y_{w,0}=0.22$, with $RH_{\text{ext}}=40\text{\%}$ and $p_{\text{ext}}=p_{0}$, while varying the external temperature $T_{\text{ext}}$. These initial conditions are chosen, because they are representative for expanding starchy snacks (Van der Sman and Broeze, 2014b, Van der Sman and Broeze, 2014a). The results are presented in the same manner as for the previous series of simulations, as shown in Fig. 8.

We observe that only for $T_{\text{ext}}/T_{\text{boil}}\geq 1.05$ does the gas pressure rise significantly above the boiling line. This behaviour can be attributed to the reduced water activity at lower moisture contents, which thus increases the boiling point above 100 ° C. However, at $T_{\text{ext}}/T_{\text{boil}}=1.05$, the outer surface enters the glassy state almost immediately, thereby suppressing puffing of the system. Stable puffing is observed in the range $1.08\leq T_{\text{ext}}/T_{\text{boil}}\leq 1.11$. For higher values of $T_{\text{ext}}/T_{\text{boil}}$, the pore growth becomes unbounded, as $T_{g}/T_{\text{ext}}\to 1.0$. These results indicate that, even at lower initial moisture content, the processing window for controlled puffing remains narrow.

**Fig. 9:**
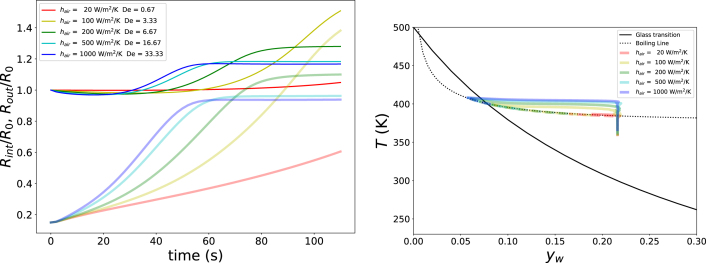
Left pane: Growth of inner (translucent lines) and outer radius (solid lines) of the unit cell as function of $h_{air}$ at $T_{ext}=408$ K, $RH_{ext}=40$%, $p_{ext}=p_{0}$, and $y_{w,0}=0.22$. The legend denotes also the value of the Deborah number at the end of simulation. Right pane: Corresponding paths of the outer surface (dashed lines) and pore wall (solid lines) in the state diagram.

In the next series of simulations, we investigate the effects of the time scales of heat transfer and viscoelastic relaxation, as captured by the Deborah number De. To this end, the heat transfer coefficient is varied over the range $20<h_{\text{air}}<1000\;\text{W}\;{\text{m}}^{-2}\;{\text{K}}^{-1}$. The corresponding results are shown in Fig. 9, which reveals a clear optimum in pore formation. When De is small, heat transfer is slow, resulting in a gradual build-up of gas pressure, while elastic stresses relax rapidly. Under these conditions, pore growth is slow and may, in principle, become unbounded, but in practice this would not occur within realistic processing times - which is in the order of minutes in case of expanded snacks frying. Conversely, when De is large, the matrix enters the glassy state at an early stage, leading to only limited expansion of the system. At intermediate values, around De≈5, gas pressure builds up sufficiently rapidly, while viscoelastic relaxation remains slow enough to allow pore growth. In this regime, the pore reaches a steady, finite volume within practical processing times.

The corresponding trajectories in the starch state diagram of Fig. 9 confirm this interpretation. For small values of $h_{\text{air}}$, little gas pressure is generated, whereas for large $h_{\text{air}}$ the system rapidly transitions into the glassy state. For intermediate values of $h_{\text{air}}$, substantial gas pressure develops, and the outer shell enters the glassy state only towards the end of the process, thereby stabilising the pore structure.

**Fig. 10:**
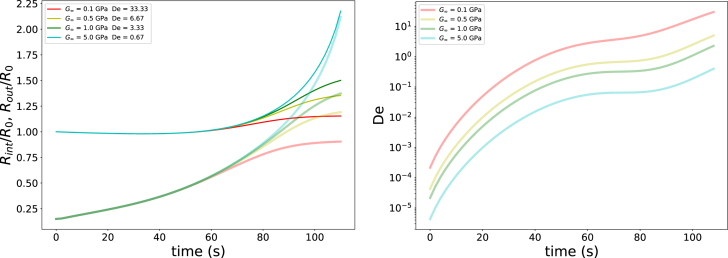
Left pane: Growth of inner (translucent lines) and outer radius (solid lines) of the unit cell as function of $G_{\infty }$ for $h_{air}$=100 W/m${}^{2}$ K, $T_{ext}=408$ K, $RH_{ext}=40$%, $p_{ext}=p_{0}$, and $y_{w,0}=0.22$. The legend denotes also the value of the Deborah number at the end of simulation. Right pane: Development of the Deborah number over time for various $G_{\infty }$.

In the final series of simulations, we vary the elastic modulus in the glassy state, $G_{\infty }$, in order to modify the viscoelastic relaxation time $\tau_{\text{visco}}$ without altering the characteristic time scales for heat and moisture transfer. The modulus is varied over the range $0.1<G_{\infty }<50\;\text{GPa}$, corresponding to $10<\tau_{\text{visco}}<500\;\text{s}$ at $T_{g}/T=1$. The processing time $t_{e}$ lies within this range. The results are shown in Fig. 10. Because the heat and mass transfer rates are identical in all simulations, the trajectory of the product through the state diagram is unchanged. For this reason, the corresponding state-diagram paths are not shown.

Fig. 10 shows that pore expansion increases with decreasing Deborah number. However, for De<3, the pore growth becomes unbounded, which would ultimately lead to structural collapse. At high Deborah numbers, only limited expansion is observed. From the right pane of Fig. 10, it follows that pore growth initiates for $De≳10^{-2}$, while stabilised pore growth is obtained for De∼3.

These trends are in good agreement with the results from the previous series of simulations in which the heat transfer coefficient $h_{\text{ext}}$ was varied. We therefore conclude that the Deborah number is a key governing parameter for pore growth and stabilisation in puffing processes. We remind the reader that many different factor govern De, as indicated by Eq. (38).

## 4 Discussion

Via usage of a multiphysics model, describing heat and mass transfer couple to large deformations, we simulated pore formation during intensive heating. With the model, describing a spherical unit cell consisting of an initially small pore surrounded by a starchy matrix, we have identified the key variables governing pore growth and crust formation. Pore formation occurs when the external temperature exceeds the boiling temperature, i.e. when $T_{\text{ext}}/T_{\text{boil}}>1$. However, the formation of a *stable* pore requires the rapid development of a glassy crust, corresponding to $T_{g}/T_{\text{ext}}>1$. In addition to these thermodynamic conditions, dynamic effects (as captured by De) play a crucial role in determining the final pore structure.

When the initial moisture content $y_{w,0}$ is high, removal of water from the matrix and crust proceeds slowly. Under such conditions, the pore may undergo unbounded growth if $T_{\text{ext}}/T_{\text{boil}}>1$ while $T_{g}/T_{\text{ext}}<1$. In the simulations, this leads to numerical divergence; in practice, such uncontrolled growth would result in rupture of the pore wall followed by structural collapse. A useful indicator for the pore growth dynamics is the Deborah number, which expresses the ratio between the viscoelastic relaxation time and the characteristic time scale for moisture removal during intensive heating. Overall, pore formation is governed by the interplay between processing conditions ($T_{\text{ext}}$, $RH_{\text{ext}}$, $p_{\text{ext}}$) and material properties, as reflected by the initial moisture content $y_{w,0}$ and the glass transition temperature $T_{g}$.

**Fig. 11:**
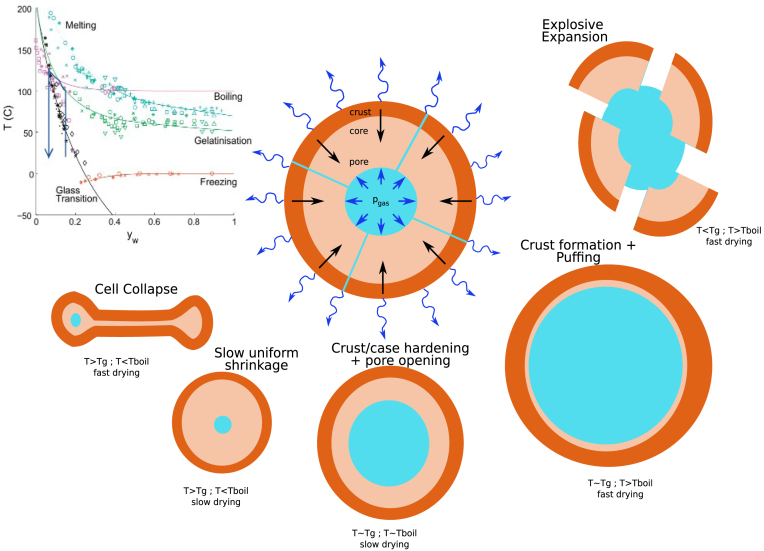
Summary of different outcomes of pore expansion processes as function of $T_{ext}/T_{boil}$, $T_{g}/T_{ext}$, and drying rate (relative to viscoelastic relaxation times). The state diagram indicated how glass transition and boiling temperature depends on moisture, which is theoretically derived in Van der Sman and Meinders (2011).

The pore formation process can be conveniently interpreted by tracing the trajectory of the biopolymer matrix state in the state diagram, which displays the glass transition and boiling lines as functions of moisture content. The condition $T_{\text{ext}}/T_{\text{boil}}>1$ is reflected by the path of the inner surface of the matrix — being in equilibrium with the gas bubble — crossing above the boiling line, indicating that the gas pressure exceeds the ambient pressure ($p_{\text{gas}}>p_{0}$) and thus provides a driving force for pore expansion. Stable pore growth requires that the outer surface of the matrix, i.e. the crust, approaches or enters the glassy state ($T_{g}/T_{\text{ext}}>1$). Crucially, the timing of this transition is controlled by the Deborah number, as shown by Fig. 9, Fig. 10 . If the crust becomes glassy too rapidly, expansion is arrested prematurely, whereas if the crust remains rubbery for too long, the pore grows uncontrollably and ultimately ruptures and collapses. In Fig. 11 we have depicted the different outcomes of the pore expansion process as function of the conditions discussed above.

Our findings are well in line with experimental findings: pore expansion happens if heated above the glass transition, and pores are stabilised by glassy state, after steam escape via pore opening (He et al., 2023). For expansion a glassy skin/curst must be formed first (Pompe et al., 2020). The crust must be small compared to the product size, to enable expansion (Pompe et al., 2020). Also, the generation of vapour (over) pressure is acknowledged for pore expansion (Pompe et al., 2020). This overpressure is generated if the product temperature exceed boiling point (Miś et al., 2016). The role of the Deborah number is little discussed in food science, with exception of Achanta et al. (1997). In chemical engineering its influence is more discussed (Everitt et al., 2006, Tammaro et al., 2022), clearly stating that bubble rupture occurs if De≫1—which is inline with our findings. In chemical engineering often other gases then steam are used for blowing agents, and dissolution of the blowing agent does not bring the foam in the glassy state. This behaviour of blowing agent is more resembling CO2, which is used as extra blowing agent in foods as during break baking or biscuit/cake leavening.

## 5 Conclusions

This paper identified the prerequisites for pore formation—namely $T_{\text{ext}}/T_{\text{boil}}>1$, $T_{g}/T_{\text{ext}}>1$, and De≈1. They provide useful criteria for the assessment and optimisation of alternative oil-free frying and drying processes. Examples include air frying, microwave-assisted vacuum drying, and superheated steam drying. The applicability of these criteria to the evaluation of such processes will be the subject of future research.

The presented cell model can be a stepping stone for a full blown mesoscale, or multiscale model, which can incorporate gas bubble opening, (partial) coalescence, and disproportionation, cf. (Everitt et al., 2006, Mitrias et al., 2019, Ataei et al., 2021, Tammaro et al., 2022).

## Declaration of competing interest

Author declares no conflict of interest
